# PCSK9 and Breast Cancer Survival: A Mendelian Randomization Study

**DOI:** 10.1158/1055-9965.EPI-25-1569

**Published:** 2026-03-23

**Authors:** Janne Pott, Amy M. Mason, Ville Salo, Johannes Kettunen, for FINNGEN, Stephen Burgess

**Affiliations:** 1 MRC Biostatistics Unit, University of Cambridge, Cambridge, United Kingdom.; 2 British Heart Foundation Cardiovascular Epidemiology Unit, Department of Public Health and Primary Care, University of Cambridge, Cambridge, United Kingdom.; 3 Victor Phillip Dahdaleh Heart and Lung Research Institute, University of Cambridge, Cambridge, United Kingdom.; 4 Research unit of Population Health, Faculty of Medicine, and Biocenter Oulu, University of Oulu, Oulu, Finland.; 5 Medical Research Center Oulu, Oulu University Hospital, University of Oulu, Oulu, Finland.

## Abstract

**Background:**

Proprotein convertase subtilisin/kexin type 9 (PCSK9) affects lipid metabolism. A recent study identified an association between rs562556 within the *PCSK9* gene and breast cancer survival (BCS), suggesting PCSK9 inhibition as an early intervention strategy to prevent metastasizing breast cancer. We attempt to replicate these findings using genome-wide association study (GWAS) data and Mendelian Randomization (MR).

**Methods:**

We used GWAS data from the Breast Cancer Association Consortium (BCAC, *N* = 91,686) and performed analyses in the FinnGen study (*N* = 4,648; additive and recessive model). First, we tested the association of the single variant rs562556, then genetically proxied PCSK9 inhibition using multiple variants, and finally adjusted for the indirect effects of lipid metabolism using multivariable MR. Coronary artery disease was chosen as a positive control outcome.

**Results:**

Estimates are scaled as log hazard ratios (logHR) per 1 SD higher PCSK9 levels. We found no significant association between *PCSK9* variants and BCS in any MR approach with any replication data: single variant [BCAC: logHR = 2.37, *P* = 0.066; FinnGen additive: logHR = 0.58, *P* = 0.91; FinnGen recessive: logHR = −2.75, *P* = 0.87], multiple variants [BCAC: logHR = −0.22, *P* = 0.30; FinnGen: logHR = −0.40, *P* = 0.54], or multivariable [BCAC: logHR = 0.42, *P* = 0.65; FinnGen: logHR = 1.23, *P* = 0.75]. Positive control analyses were significant throughout.

**Conclusions:**

A significant association of *PCSK9* variants with BCS could only be reproduced using outcome data from the original study but not when using independent, larger GWASs.

**Impact:**

Potential reasons for the discrepant results are different genetic models, sample selection criteria, and the time variability of HR estimates. They should be explored before considering PCSK9, a therapeutic target in patients with breast cancer.

## Introduction

**Figure 1.**
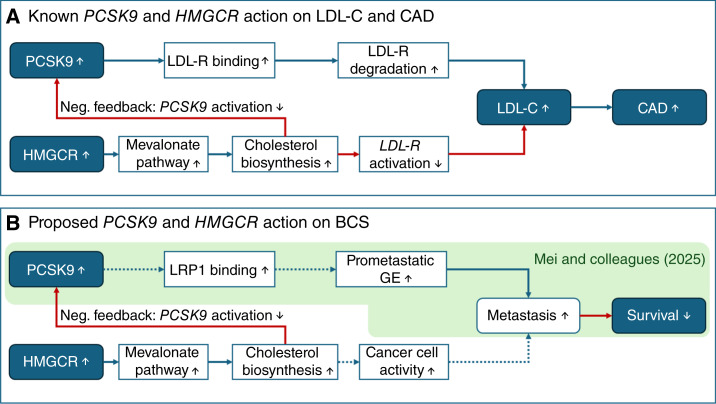
Directed acyclic graphs. Blue filled boxes indicate considered exposures and outcomes in this study. Blue arrows indicate a positive association (e.g., higher LDL-C levels lead to higher CAD risk), whereas red arrows indicate negative associations (e.g., less *LDLR* activation leads to higher LDL-C levels in blood). Solid lines indicate established links, whereas dashed lines indicate links that have been suggested but not yet validated. **A,** Classic path of action for PCSK9 and LDL-C levels on risk for CAD. PCSK9 binds to LDL-R and leads to its degradation, resulting in higher LDL-C levels. HMG-CoA activity activates intracellular cholesterol biosynthesis, resulting in reduced LDL-R expression. LDL-R expression is regulated by a shared transcription factor of PCSK9. Hence, PCSK9 levels are also reduced. **B,** Considered the path of action of PCSK9 on BCS in this work. Mei and colleagues (15) reported higher levels of PCSK9 associated with BCS, presumably due to its binding to LRP1, which in turn leads to increased gene expression of prometastatic genes. The induced metastases are then the cause of the reduced survival. It has been suggested that HMG-CoA affects metastasis risk by its effect on cholesterol and cancer cell activity (11, 12).

Proprotein convertase subtilisin/kexin type 9 (PCSK9) is mainly expressed in the liver and plays a key role in lipid metabolism (1). It binds to low-density lipoprotein receptors (LDL-R) on the surface of hepatocytes and prohibits LDL-R recycling after its internalization (2). Hence, PCSK9 leads to LDL-R degradation and higher levels of LDL cholesterol (LDL-C) in the blood (see Fig. 1A). PCSK9’s pivotal role in LDL-C clearance has led to the development of various PCSK9 inhibitors to treat hypercholesterolemia (3–5), which are usually given when other lipid-lowering medications such as statins are not tolerated or fail to achieve the target reduction of LDL-C levels.

Statins are currently the primary therapeutic modality for the management of hyperlipidemia. They inhibit 3-hydroxy-3-methylglutaryl-coA (HMG-CoA) reductase (encoded by *HMGCR*) and reduce LDL-C levels by decreasing intracellular cholesterol synthesis via the mevalonate pathway. In response to the lower intracellular cholesterol level, the gene expression of LDL-R is activated. However, the same transcription factor activating *LDLR* also regulates *PCSK9* (see Fig. 1A). As a result, correcting for this interaction between *PCSK9* and *HMGCR* is essential for understanding the direct impact of PCSK9 on any other complex outcome, independent of PCSK9’s effect via LDL-C.

One example of a complex outcome affected by PCSK9 and LDL-C is coronary artery disease (CAD), and both statins and PCSK9 inhibitors are used in CAD prevention (6, 7). Statins have been considered for other outcomes such as breast cancer risk, for which mixed evidence has been reported: on the one hand, a meta-analysis of 2.4 million participants and 76,759 patients found no significant protective effect of statins against breast cancer (8). On the other hand, Mendelian Randomization (MR) studies tested the effects of statins on breast cancer risk by instrumenting variants at *HMGCR* associated with LDL-C (9, 10) and found associations between genetically proxied inhibition of HMG-CoA reductase and reduced breast cancer risk. However, there were no significant associations between variants at *PCSK9* or other lipid-lowering treatment targets and breast cancer risk or between genetically predicted LDL-C and breast cancer risk.

The effect of statins and cholesterol levels has also been studied in the context of breast cancer survival (BCS) in patients with breast cancer. A clinical trial analyzing disease-free survival in patients with premenopausal, stage I and II breast cancer found improved survival in patients additionally taking zoledronic acid (11), which, like statins, inhibit the mevalonate pathway. Trials testing statins for breast cancer survival are still underway (see Fig. 1B; ref. 12). PCSK9 has also been considered in breast cancer survival research. It binds not only to LDL-R but also to LDL-R–related protein 1 (LRP1), among others (13). It has been shown that LDL-R effectively competes with LRP1 for PCSK9 activity (14), and host PCSK9 binding to tumoral LRP1 was suggested to affect metastatic colonization by activating prometastatic gene expression (15). Others have already shown that the lack of host PCSK9 reduced metastasis in the liver in mice (16), although this effect was lost after feeding the mice a high-cholesterol diet. This suggests that high cholesterol promoted metastatic progression, and there was no effect of PCSK9 independent of cholesterol. Another study analyzed PCSK9 in pancreatic cancer, in which it seemed to guide the metastatic colonization in the lung or liver due to its regulation of the cholesterol synthesis pathway (17).

A recent study by Mei and colleagues (15) used data from four well-annotated breast cancer cohorts, namely The Cancer Genome Atlas Breast Invasive Carcinoma (TCGA-BRCA), Bertucci and colleagues (18), Hartwig Medical Foundation (HMF) cohort, and Nik-Zainal and colleagues (19), of which two (HMF and Bertucci and colleagues) are metastatic breast cancer cohorts. For the other two cohorts, Mei and colleagues (15) selected high-risk patients with stage II or III breast cancer who were 50 years and older at diagnosis. They identified a missense mutation in the *PCSK9* gene, namely rs562556, to be associated with breast cancer metastasis and survival. In more detail, Mei and colleagues (15) used a recessive single-nucleotide polymorphism (SNP) model to test for an association between the rs562556 major allele A and BCS and observed significant associations in TCGA-BRCA data, Bertucci and colleagues (18), and Nik-Zainal and colleagues (19). In the HMF cohort, the association did not reach significance. Restricted to European ancestry, the association between rs562556 and breast cancer prognosis was still significant in the two larger cohorts, TCGA-BRCA and Bertucci and colleagues (18). In contrast to others (16, 17), Mei and colleagues (15) found this PCSK9 mechanism not to be mediated by LDL-C reduction and suggested that targeting homozygous carriers of the major allele of rs562556 might allow for early intervention strategies to prevent metastasizing breast cancer in patients (see Fig. 1B).

In this study, we explore the link between PCSK9 and BCS further using three MR approaches, with the main aim of replicating the following findings of Mei and colleagues (15):

1) Single variant (rs562556, total effect): To assess the causal role of the missense mutation rs562556, we extracted its summary statistics from Mei and colleagues (15), Morra and colleagues (20) [data from the Breast Cancer Association Consortium (BCAC)], and the FinnGen cohort (21) and then used the MR ratio method, combining publicly available exposure data (22–25) and these BCS association statistics to make causal inferences about the functionality.
2) Multiple variants (total effect): To assess the total effect of PCSK9 inhibition, we performed classic two-sample MR approaches using publicly available *PCSK9 *instruments (22–25) and matched statistics from Morra and colleagues (20) and FinnGen (21).
3) Multivariable approach (direct effect): To assess the independence of the PCSK9 effect from lipid metabolism, we performed three-sample multivariable MR (MVMR) using PCSK9 (22), LDL-C (24, 25), and BCS data (20, 21), hence estimating the direct PCSK9 effect while adjusting for potential mediation via LDL-C.

All three replication attempts were repeated for CAD as a positive control (known effect of *PCSK9* SNPs on CAD risk via lipid metabolism) and for breast cancer risk as a negative control (so far, no evidence that *PCSK9* variants affect breast cancer risk).

## Materials and Methods

### Data sources

The analysis plan with data sources is displayed in Supplementary Fig. S1. Publicly available genetic association summary statistics were used for most analyses. Links for data access are provided in the data availability statement. Appropriate ethical approvals and participant consent were obtained from the original studies that generated the data. We used the R Project for Statistical Computing (RRID: SCR_001905) v4.2.2 for all data analyses.

#### Exposure data

The primary exposure was PCSK9 protein levels in blood, as reported in our previous work on a sex-stratified meta-genome-wide association study (GWAS) in Europeans (22). As BCS is female-specific, we also focused on PCSK9 in females (*n* = 8,936) and used the sex-combined data as a sensitivity check (*n* = 20,016). The data for males and females were combined using the R package *meta* (RRID: SCR_019055; ref. 26).

As a secondary exposure, we also obtained significant genetic association estimates on PCSK9 gene expression levels from GTEx v10 (23), which were available in 16 tissues. We excluded the tissue testis, as this male-specific tissue is not relevant for the female-specific outcomes considered here. To test and correct for LDL-C levels affecting BCS, we downloaded both the female-specific and sex-combined GWAS summary statistics from the Global Lipids Genetics Consortium (GLGC; Europeans only, maximum sample size females = 558,500; sex-combined = 1,231,284), as published in Kanoni and colleagues (25) and Graham and colleagues (24), respectively.

#### Primary outcome data

The primary outcome was BCS, and data for this outcome were available in three studies. First, we used GWAS summary statistics from the BCAC, as published in Morra and colleagues (20). They analyzed the survival of 91,686 patients with breast cancer of European ancestry, with 7,531 breast cancer–specific deaths. Patients were above 18 years of age at diagnosis with any stage of breast cancer. In their GWAS, they used Cox regression to estimate the 15-year hazard ratios (HR) in an additive allele effect model.

Second, we used the SNP rs562556 summary statistics from Mei and colleagues (15), as provided in their supplementary Fig. S1H (HR and *P* values of rs562556 association with BCS in patients of European ancestry). This comprised four cohorts: TCGA-BRCA, Bertucci and colleagues (18), Nik-Zainal and colleagues (19), and the HMF cohort. No sample size information or case numbers were reported for the European subsets. In two cohorts, patients were restricted to females above 50 years of age at diagnosis with stage II or III breast cancer, hence enriched in patients with a high risk of metastasis [TCGA-BRCA and Nik-Zainal and colleagues (19)]. Here, survival was censored at 10 years. In the other two studies, patients had metastatic breast cancer, and survival time was censored at 2 years [HMF cohort and Bertucci and colleagues (18)]. In their SNP tests per study, Mei and colleagues (15) used Cox proportional hazards regression with a recessive allele effect model. Here, we meta-analyzed the four reported HRs using the R package *meta* (RRID: SCR_019055; ref. 26).

Given the differences in sample selection and tested SNPs, we obtained data from the FinnGen study (RRID: SCR_022254; ref. 21), using the selection criteria from Mei and colleagues (15) for TCGA-BRCA and testing all possible instruments for association. The FinnGen study is a large-scale genomics initiative that has analyzed more than 500,000 Finnish biobank samples and correlated genetic variation with health data to understand disease mechanisms and predispositions. The project is a collaboration between research organizations and biobanks within Finland and international industry partners (21). The FinnGen project possesses the ethical and prior permits required for biobank research (see Supplementary Note S1). All participants have provided written consent to biobank research, either in connection with sample donation or when taking part in previous research projects in which materials have been transferred to Finnish biobanks with the approval of Fimea, the National Supervisory Authority for Welfare and Health. Information on genotyping and imputation can be found in the Supplementary Note. The FinnGen data freeze R12 was utilized, and female patients with breast cancer were identified based on the International Classification of Diseases, 10th Revision (ICD-10) code C50. Only patients who had stage II or stage III breast cancer and who were older than 50 at the time of diagnosis were included in the analysis. The final study sample consisted of 4,648 patients with breast cancer.

#### Secondary outcome data

We repeated the MR analyses on positive and negative controls. As a negative control, we used breast cancer risk, as current literature does not link PCSK9 or LDL-C reduction by PCSK9 inhibition to breast cancer risk. Data were taken from the FinnGen and UK Biobank (UKB, RRID: SCR_012815) meta-analysis (cases = 30,593 and controls = 388,840; ref. 21), and we expected to find a null result here. As a positive control, we used CAD (data source: Aragam and colleagues (27); meta-analysis of CARDIoGRAMplusC4D, UKB, and nine other studies of European ancestry). CAD summary statistics were available both sex-combined and for females only (cases = 181,522; controls = 984,168 in the sex-combined setting). PCSK9 and LDL-C affect survival by increasing the risk for cardiovascular events. Hence, increasing levels of PCSK9 or LDL-C are expected to have a significant positive effect on CAD.

### Instrumental variables

#### Replication 1: single variant approach

For the single variant approach, we focused on rs562556, the proposed causal SNP from Mei and colleagues (15). For PCSK9 protein levels, we used data from Pott and colleagues (22), and the association reached genome-wide significance in the sex-combined setting but not in females only ($P=5.33\times 10^{-5}$). For gene expression of PCSK9, we used data from the GTEx consortium (v10, RRID: SCR_001618; ref. 23). The SNP was a significant expression quantitative trait locus (eQTL) in two tissues: spleen and esophagus muscularis. Finally, for LDL-C, we used data from GLGC (24, 25). In all three datasets, an additive SNP model was used. We harmonized the alleles and used the minor allele G as the effect allele here.

#### Replication 2: multiple variants approach

For PCSK9 protein levels in the multiple variants approach, we selected the same four SNPs as described in Pott and colleagues (22), namely rs11591147, rs693668, rs11583680, and rs2495491. They all reached genome-wide significance ($P<5\times 10^{-8}$) and are pairwise independent (LD $r^{2}<0.1$).

For gene expression of PCSK9, we filtered the GTEx v10 data (23) for genome-wide significant associations. Position information was given in hg38, and we lifted the position information down to hg19 by matching the rsID to the LDL-C dataset. Hence, we excluded eQTLs with missing reference SNP ID (rsID) or no matching alleles in eQTLs and LDL-C data. This left us with eight tissues with genome-wide significant eQTLs (adipose visceral omentum, brain cerebellar hemisphere, brain cerebellum, lung, tibial nerve, pancreas, spleen, and whole blood). For each tissue, we then selected independent variants after clumping to a pairwise LD threshold of $r^{2}<0.1$ using the 1000 Genomes European reference panel assessed with Functional Mapping and Annotation of GWASs (RRID: SCR_017521; ref. 28).

For LDL-C, we restricted the GLGC data (24, 25) to genome-wide significant SNPs after genomic control correction, minor allele frequency > 0.01, and annotated with rsID. We further reduced the variants to those also available in the outcome datasets. We then used a position-based priority pruning approach, in which we selected the best-associated (highest absolute *z*-score) variant as the index SNP and excluded all variants within ± 500 KB of the index SNP as tagged SNPs. Finally, we only used index SNPs with strong support by excluding those with less than nine tagged SNPs. This choice of threshold resulted in 171 and 269 independent index SNPs for the MR analyses for females and the sex-combined setting, respectively. In addition to the genetically proxied LDL-C levels, we also selected instruments to proxy HMG-CoA reductase or PCSK9 inhibition. For this, we used independent SNPs at *HMGCR* and *PCSK9* as reported by Yang and colleagues (eTable 3; ref. 29).

We harmonized the effect allele to be the minor allele throughout and excluded triallelic SNPs and SNPs with an effect allele frequency difference >0.1 between exposure and outcome. A total of 405 SNPs were selected, pairwise independent for their respective exposure.

#### Replication 3: multivariable approach

For the multivariable approach, we used two different strategies to select instruments for PCSK9 and LDL-C. First, we selected the four variants described in Pott and colleagues (22). These four SNPs were available in all relevant datasets. Given the high correlation between the SNP associations with PCSK9 and LDL-C, we added a sensitivity approach with more instruments from a different gene region, namely *HMGCR*. We used the independent SNPs as reported by Yang and colleagues (Supplementary Table S3; ref. 29) and restricted our analysis to significant associations in at least one exposure.

### FinnGen genotyping and statistical analysis

Cox regression with an additive model was utilized to estimate HR and 95% confidence intervals for 403 predefined SNPs, with age used as a covariate. Two variants, namely rs11690912 and rs10206674, were missing in FinnGen because of quality control (QC) exclusions (see Supplementary Note S1). In addition, we performed a Cox regression with a recessive model for variant rs562556 using age as a covariate, as in our main analysis. The time to event was censored at the time of last follow-up or at 10 years after diagnosis, whichever came first. Patients who died from causes other than breast cancer were censored at the time of death if death occurred before 10 years from diagnosis or at 10 years if it did not. A total of 288 breast cancer deaths occurred among 4,648 patients with the disease.

For five SNPs, the allele frequency (AF) was very low in the FinnGen study (AF < 0.01). Hence, we excluded those five variants. All 398 used instruments and their summary statistics on exposures and outcomes are found in the Supplementary Tables S1 (exposure association for the MR approach), S2 (exposure association for the MVMR approach), and S3 (outcome association for both MR and MVMR approaches).

### MR

We tested for causal effects of PCSK9 and LDL-C levels on BCS and all secondary outcomes (breast cancer and CAD risk) using three approaches. For all approaches, we used a significance threshold of α=0.05, and corrected for multiple testing using the Benjamini–Hochberg procedure (30).

#### Replication 1: single variant approach

First, we estimated the Wald ratio and standard error from the first term of the delta method (31) using the SNP rs562556, as it was suggested as a causal variant for BCS.

#### Replication 2: multiple variants approach

Second, we combined the available instruments per sex subgroup or tissue using inverse variance weighted MR (MR-IVW). The method was used as implemented in the R package *MendelianRandomization* (RRID: SCR_025049) v0.10.0 (32). When only one instrument was available, the simple ratio and standard error from the first two terms of the delta method were estimated (31). For LDL-C, we ran the MR-IVW using either any available instrument, SNPs at *PCSK9 *to mimic PCSK9 inhibition, SNPs at *HMGCR *to mimic statin treatment, or SNPs at both *PCSK9 *and *HMGCR*.

#### Replication 3: multivariable approach

In a third approach, we estimate the direct PCSK9 effect conditional on the LDL-C effect using MVMR. The MVMR-IVW was performed using the same R package, and we used the maximum sample size per exposure to estimate the conditional F-statistics. As the main analysis, we used four instruments at *PCSK9* as reported by Pott and colleagues (22). As a sensitivity analysis, we used SNPs at *PCSK9* and *HMGCR* from the LDL-C approach, strengthening the MVMR by including more instruments. Other combinations, such as all valid LDL-C variants, would result in weak instruments for PCSK9.

## Results

### Replication 1: single variant approach

In a first step, we meta-analyzed the data of the original study (15) to obtain a pooled estimate of the genetic association based on well-established breast cancer cohorts. Although the four studies are heterogeneous about their selection criteria (metastatic or nonmetastatic), we detected no sign of heterogeneity in this meta-analysis (Cochran’s Q: 3.51, *P* value of heterogeneity: 0.319 with 3 degrees of freedom). A forest plot of this meta-analysis can be found in Supplementary Fig. S2. We then looked up this SNP in the publicly available Morra and colleagues (20) dataset and additionally analyzed it in FinnGen (21), using both a recessive and additive model. An overview of the used outcome data and their reported association statistics is given in Table 1. The association did not reach significance in neither Morra and colleagues (P=0.066; ref. 20) nor FinnGen (P=0.91 and P=0.87 for the additive and recessive genetic model, respectively; ref. 21).

**Table 1.** Overview of used outcome data sources for BCS.

Data	Sample selection	SNP model	No. of samples (no. of deaths)	LogHR [SE(logHR)]	*P* value
Original study of Mei and colleagues (15)					​
HMF cohort	Metastatic breast cancer 2-year HR	Recessive	<283	−0.30 (0.16)	0.058
Bertucci and colleagues (18)			<553	−0.39 (0.14)	**0.0045**
Nik-Zainal and colleagues (19)	50+ at breast cancer diagnosis Stage II or III 10-year HR	Recessive	<101	−2.05 (1.05)	0.051
TGCA-BRCA			<519	−0.63 (0.29)	**0.028**
Meta-analysis of Mei and colleagues (15) data					​
Fixed-effect		Recessive	<1,456	−0.40 (0.10)	**4.1 × 10** ^**−5**^
Replication cohorts					​
FinnGen (21)	50+ at breast cancer diagnosis Stage II or III 10-year HR	Recessive Additive	4,648 (288)	+0.06 (0.36) −0.01 (0.11)	0.87 0.91
Morra and colleagues (20)	18+ at breast cancer diagnosis Any stage of breast cancer 15-year HR	Additive	91,686 (7,531)	−0.05 (0.03)	0.066

NOTE: The original study of Mei and colleagues (15) reported four small breast cancer cohorts testing the *PCSK9* variant rs562556 in the recessive model only. We report here the fixed-effects pooled estimate of these four cohorts. The sample selection criteria of two of these cohorts (Nik-Zainal and colleagues and TCGA-BRCA) were used to select patients with breast cancer in the FinnGen (21) study to allow a replication approach most similar to Mei and colleagues (15). Morra and colleagues (BCAC; ref. 20) used a genome-wide approach and different sample selection. The logHR and *P* values of the rs562556 association for BCS are given per minor allele G in the additive models, and for genotyes G/G or A/G in the recessive model. Please note that due to the allele switch to harmonize all datasets, we now technically report the dominant model for allele G, which is the negative recessive model for allele A. Bold text indicates *P* values significant associations (*P* < 0.05).

**Figure 2.**
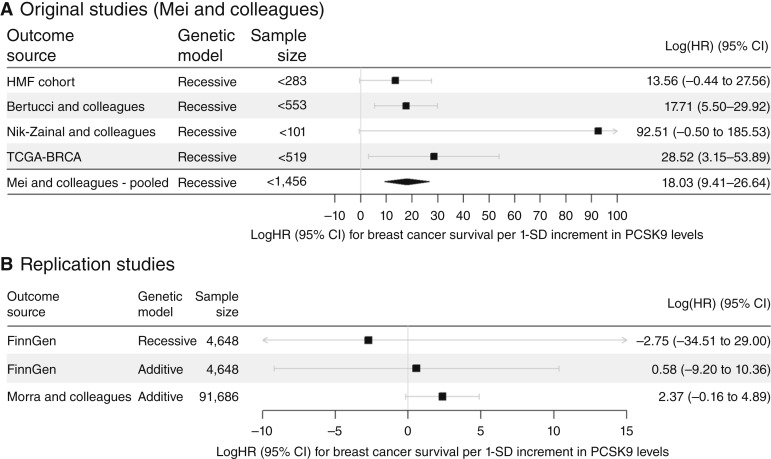
Forest plot of the single variant analyses of PCSK9 levels, proxied by rs562556, on BCS. The logHR for BCS per 1 SD increment in PCSK9 levels is given for protein expression in females. **A,** MR ratio results when using the outcome data as reported by Mei and colleagues (Supplementary Fig. S1H; ref. 15) and when using the fixed-effect meta-analysis estimate for the outcome. After correcting for multiple testing, the effect using the Bertucci and colleagues (18) study and the pooled estimate was significant. Please note: the exact sample size per study for Europeans only was not reported. Hence, we provide here the sample sizes reported in Mei and colleagues (15) main analysis (Supplementary Fig. S1C–S1F). **B,** MR ratio results when using the replication studies FinnGen (21), either using the recessive or additive SNP model, and Morra and colleagues (BCAC; ref. 20). CI, confidence interval.

We then used the single variant rs562556 in the MR ratio approach to test for significant association between PCSK9 levels and BCS, using all either the four single study results of the original publication [Mei and colleagues (15), recessive SNP effect], the pooled estimate of the four single studies (see Fig. 2A), the recessive and additive estimate from FinnGen (21), or the additive estimate from Morra and colleagues (see Fig. 2B; ref. 20). Significant exposure associations were available for PCSK9 protein levels (females only and sex-combined) and *PCSK9* gene expression in spleen and esophagus (muscularis) tissue, with F-statistics >10 (16.3, 63.7, 20, and 18.9, respectively). We observed a significant positive association between PCSK9 levels and BCS when using the pooled estimate of the original study data [logHR (logHR) per 1 SD higher PCSK9 in females: logHR=18.03, $P=4.1\times 10^{-5}$] but not in the replication (see Table 2). With the Morra and colleagues (20) data, we estimated a logHR of 2.37 (P=0.066). Using the additive genetic model in FinnGen (21), we observed a logHR of 0.58 (P=0.91), whereas the recessive model was estimated as logHR=-2.75 (P=0.87). The same was true when using sex-combined PCSK9 data, PCSK9 gene expression (see Supplementary Fig. S3), or LDL-C data (see Supplementary Fig. S4). All results are summarized in Supplementary Table S4. We note that the estimates are unrealistically high when using the original study data and should not be overinterpreted. Also, the analyses using Mei and colleagues’ data (15) are not independent, nor are these true replications of the previously published findings, as they are based on the same outcome associations.

**Table 2.** Overview of the MR results of PCSK9 and LDL-C levels on BCS in females.

MR method	Source	PCSK9 on BCS			LDL-C on BCS		
		LogHR	*P*	F	LogHR	*P*	F
Single variant rs562556	Pooled data from Mei and colleagues (recessive; ref. 15)	**18.03**	**4.1 × 10** ^**−5**^	16.3	**10.93**	**4.1 × 10** ^**−5**^	188
	FinnGen (recessive; ref. 21)	−2.75	0.87		−1.67	0.87	
	FinnGen (21)	0.58	0.91		0.35	0.91	
	Morra and colleagues (20)	2.37	0.066		1.44	0.066	
Multiple variants	FinnGen (21)	−0.40	0.54	121	−0.14	0.75	483
	Morra and colleagues (20)	−0.22	0.30		−0.23	0.070	
Multivariable approach	FinnGen (21)	1.23	0.75	9.2	−1.24	0.67	9.3
	Morra and colleagues (20)	0.42	0.65	​	−0.50	0.48	​

NOTE: The logHR and *P* values for BCS per 1 SD higher PCSK9 or LDL-C are given. In the MR-ratio method, only rs562556 within *PCSK9* was used. Mei and colleagues (15) indicate the results from the pooled analysis of the four cohorts reported there. In the multiple variants approach, we used MR-IVW and instruments at *PCSK9*. In both the single and multiple variants approaches, PCSK9 and LDL-C were tested separately. In the multivariable approach, PCSK9 and LDL-C were used together on the same outcome data, using instruments at *PCSK9*. Bold entries indicate significant estimates after correcting for multiple testing. Results of the sex-combined analyses can be found in Figs. 3 and 4 and in the Supplementary Tables S4–S6.

Abbreviations: F, (conditional) F-statistic, fix for each approach; P, *P* value.

### Replication 2: multiple variant approach

**Figure 3.**
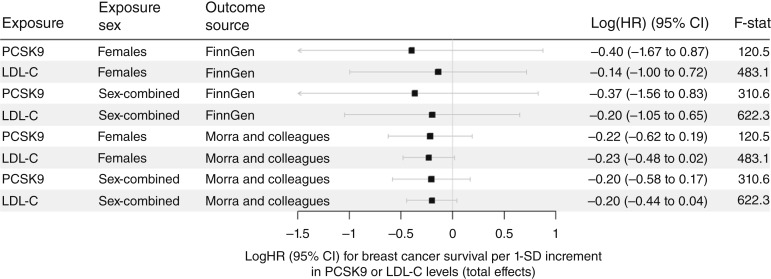
Forest plot of the multiple variant analysis of PCSK9 and LDL-C levels on BCS. We report here the logHR of the MR-IVW analysis for BCS per 1 SD increment in PCSK9 or LDL-C levels using both replication studies. Only instruments in the *PCSK9* gene region were considered. In this approach, the total effects were estimated separately for PCSK9 and LDL-C. None of the estimates were significant. The results for females are also summarized in Table 2; for further information, see Supplementary Table S5. CI, confidence interval.

Mei and colleagues (15) also reported a significant reduction in metastasis due to PCSK9 inhibition. To replicate these findings, we used multiple independent variants at the *PCSK9* gene region associated with PCSK9 protein levels or gene expression. Here, outcome data were only available in FinnGen (21) and Morra and colleagues (20). The analysis was well powered again, with F-statistics ranging from 32.1 (gene expression in pancreatic tissue) to 622.3 (LDL-C level sex-combined). The results are summarized for PCSK9 and LDL-C in females in Fig. 3 and Table 2**.** Again, we did not observe a significant association between genetically predicted PCSK9 levels and BCS (e.g., logHR per 1 SD higher PCSK9 in females: logHR=-0.21 in Morra and colleagues (20); logHR=-0.39 in FinnGen (21), both P>0.05, see also scatter plot in Supplementary Fig. S5A and S5B). Similarly, the effect of genetically predicted PCSK9 gene expression on BCS was not significant and showed mixed effect directions (see Supplementary Fig. S6), depending on tissue and outcome source. All results are summarized in Supplementary Table S5.

For LDL-C, we also tested approaches with any valid instrument across the genome or at the *HMGCR* gene region. When using all available instruments in the MR-IVW, we observed no significant result [e.g., logHR per 1 SD higher LDL-C in females: logHR=0.0054, P=0.88 using 168 SNPs and Morra and colleagues (20) data, see Supplementary Fig. S7]. When using instruments at the *HMGCR* gene region, we observed a significant negative association with BCS in the Morra and colleagues (20) data (e.g., LDL-C in females: logHR=-0.51, $P=4.9\times 10^{-3}$) but not in the FinnGen data (logHR=-0.11, P=0.94; ref. 21). In other words, genetically proxied inhibition of HMG-CoA reductase (e.g., statin treatment) has mixed results about survival probability in patients with breast cancer.

### Replication 3: multivariable approach

**Figure 4.**
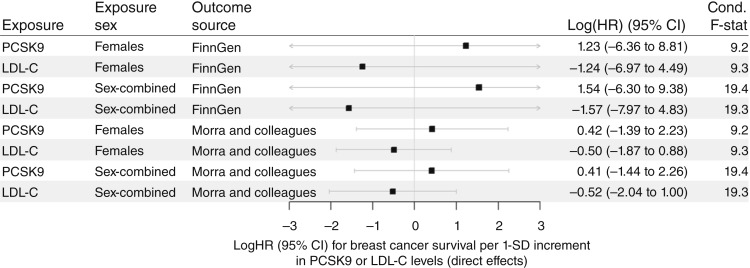
Forest plots of the multivariable analyses of PCSK9 and LDL-C conditional on each other on BCS. We report here the logHR of the MVMR-IVW analysis for BCS per 1 SD increment in PCSK9 or LDL-C levels using both replication studies. Only instruments in the *PCSK9* gene region were considered. In this approach, the direct effects were estimated, e.g., the PCSK9 effect conditional on the LDL-C effect and vice versa. None of the estimates were significant. The results for females are also summarized in Table 2; for further information, see Supplementary Table S6. CI, confidence interval.

Finally, Mei and colleagues (15) suggested that the PCSK9 effect would be independent of the effect of lipid metabolism. To test this hypothesis, we used the MVMR-IVW approach to estimate the direct effect of PCSK9 on BCS, conditional on the LDL-C effect. In Table 2, we provide a comparison of the results for LDL-C and PCSK9 per MR approach and SNP selection. Using the instruments at *PCSK9* and Morra and colleagues (20) outcome data, we found that the effect of LDL-C on BCS stayed similar to the univariable estimate for *HMGCR* but no longer significant (logHR=-0.50, P=0.48). The effect of PCSK9 changed direction but remained nonsignificant (logHR=0.42, P=0.65, conditional F-statistic = 9.2, see Fig. 4; Table 2). Using the FinnGen (21) outcome data, we observed a similar pattern, with no significant results and different effect directions. Using sex-combined exposure data did not change the results (see Supplementary Table S6 for all results).

There is a high genetic correlation at *PCSK9* between the SNP effects on PCSK9 and LDL-C, which limits the power of the MVMR approach. In a sensitivity analysis, we added variants at *HMGCR* and reran the MVMR. The result was similar, with a nonsignificant effect for PCSK9. Of note, the effect direction was heterogeneous here, with positive estimates for PCSK9 using the Morra and colleagues data and negative estimates using FinnGen data (Morra and colleagues (20): logHR=0.43, P=0.14; FinnGen (21): logHR=-0.60, P=0.67; conditional F-statistic = 11.1). Results are shown in Supplementary Fig. S8 and Table S6.

### Sensitivity checks with breast cancer risk and CAD

We repeated our analyses for two other outcomes: breast cancer and CAD risk. Breast cancer risk acted as a negative control, and indeed none of the breast cancer tests were significant, for example, no causal effect of PCSK9 protein or gene expression levels or LDL-C levels on the risk of breast cancer (see Supplementary Figs. S5C and S9; Supplementary Tables S4–S6). In the multivariable approach, the results were also not significant.

CAD risk was used as a positive control, and as expected, almost all MR analyses with sex-combined CAD data were significant with a positive effect direction (see Supplementary Figs. S5D and S10). Only the effect of PCSK9 gene expression in pancreas tissue did not reach significance (P=0.056). The effects on CAD in females were also positive, but due to lower sample size and case number, they were not always significant after multiple test correction (see Supplementary Tables S4–S6). In the multivariable approach, we were only able to detect an effect of LDL-C but not PCSK9 on sex-combined CAD (e.g., log-odds ratios (OR) per 1 SD higher PCSK9 in females: logOR=0.14, P=0.61, see Supplementary Fig. S11A). In the sensitivity test with *PCSK9* and *HMGCR* instruments, we were able to detect such an independent direct effect (logOR=0.30, P=0.013 in females, see Supplementary Fig. S11B).

## Discussion

In this study, we tried to replicate the findings of Mei and colleagues (15), who observed a significant effect of the PCSK9 gain-of-function variant rs562556 on breast cancer metastatic colonization and suggested that PCSK9 inhibition might improve survival chances in patients with breast cancer who are homozygous for the major allele of the missense mutation rs562556. We used MR to estimate causal effects of lifetime exposure to PCSK9 and tested for BCS, using data from Morra and colleagues (BCAC; ref. 20) and the FinnGen cohort (21).

Broadly speaking, our analyses fall into two categories: those performed using the largest available sample size (BCAC data) and those performed using the analysis strategy as close to the original publication as possible (FinnGen). There will naturally be a compromise between considering results based on all the available data and results based on the most relevant data. Both approaches have advantages and limitations. For our replication approach within FinnGen (21), we tried to stay as close to the sample selection criteria as reported in Mei and colleagues (15): female patients with breast cancer older than 50 years at diagnosis, with stage II or III, and looking at 10-year survival. The mortality rate in FinnGen was 6.1%, whereas the BCAC data had a mortality rate of 8.1% in a 15-year survival model of any breast cancer stage and age. The four small cohorts used in Mei and colleagues (15) might have an even higher mortality rate although it was not reported. There is a conceptual difference between a cancer-specific study and a large cohort such as FinnGen: cancer studies typically recruit patients directly, whereas in FinnGen, patients were selected using reported ICD-10 codes from health care providers. Also, breast cancer–specific death was defined by the cause of death on the death certificate, which could result in misclassifications. As long as the misclassification is random, we can still gain information from these larger numbers of noisy observations (33). Another advantage of using FinnGen was the access to individual-level data, allowing us to test both additive and recessive genetic effects as was done in BCAC (20) and Mei and colleagues (15), respectively.

PCSK9 inhibitors are administered subcutaneously and then absorbed into the bloodstream. They are mainly distributed through the circulatory system, with minimal extravascular distribution. Hence, to test if PCSK9 inhibitors could affect BCS, we used genetically predicted PCSK9 protein levels from a large meta-GWAS (22). Even with the strong power that comes from cis-effects, we observed no significant effect in the multiple variant approach, with negative point estimates. In the multivariable approach, we corrected for mediating effects through lipid metabolism and estimated the direct effect of PCSK9 on BCS. Here, the effect direction changed but still failed to reach significance. Finally, we used the reported missense mutation rs562556 in a ratio estimation approach. Here, we used the effects of rs562556 as reported in the two replication studies, BCAC (20) and FinnGen, and in the original study by Mei and colleagues (15). We detected significant positive effects of PCSK9 on BCS only when using the data from the original study, but not in the replication studies. The Mei and colleagues (15) single-study estimates and the pooled estimates are unrealistically high; for example, we estimated a logHR of 18.3. This may arise due to the winner’s curse, as the variant choice was determined in the same data as the associations estimated. When using independent rs562556 data from the BCAC (20) or FinnGen, the effect was positive but not significant. This was true for both the protein expression subgroups and the tissue-wise gene expression.

For the multiple variant approaches, we used instruments with strong effects on PCSK9 levels. Their direct effect on PCSK9 levels has been previously described by others (22) and can be explained by their role in gene expression, splicing regulation, and increased protein degradation. In contrast, the missense mutation rs562556 has not been described as a driver for PCSK9 levels but rather for its functionality. In line with this, Mei and colleagues (15) described V474I, the mutation caused by rs562556, as having a higher binding affinity to tumoral LRP1, which in turn activates prometastatic gene expression and increases the risk of metastasis. However, in their mouse and cell line models, they also showed a benefit from blocking any PCSK9 with monoclonal antibodies. Hence, the two MR approaches with their separate instruments might represent different pathways of PCSK9 action, either by levels or function. The different scaling and significance in the MR ratio estimates could be explained by several factors, which we discuss in the following sections.

First, Morra and colleagues (20) and Mei and colleagues (15) used different genetic models to estimate the effect of rs562556 on BCS: an additive allele effect model or a recessive effect model, respectively. The choice of genetic model can affect the observed SNP effect estimate size and hence the ratio estimate size. In FinnGen, both genetic models were tested, and in both tests, the effect of rs562556 was not significant on BCS. In FinnGen with the recessive model, which represents the replication of the SNP effect, we also observed a different effect direction than reported by Mei and colleagues (15).

Second, all studies used the Cox regression model to estimate the average HR of the exposed versus unexposed. For rs562556, this means a higher versus lower binding affinity of PCSK9 to LRP1, whereas for the PCSK9 QTLs, it stands for higher versus lower levels of PCSK9. However, the estimated effect size depends on the considered survival time, as the average HR ignores the distribution of events during the follow-up (34). In our case, this time variability of HR might have caused the difference in effect sizes, as Morra and colleagues (20) were looking at 15-year survival, whereas Mei and colleagues (15) considered 10-year survival for two cohorts with high-risk patients and 2-year survival for two cohorts of patients with metastasis. In addition, Mei and colleagues (15) stratified their sample sets by including only patients with a high risk for metastasis: females above 50 years of age at diagnosis with stage II or III breast cancer. This sample stratification can potentially induce bias in the HR estimates and limit generalization to low-risk patients, as included in Morra and colleagues (20), for example, females above 18 years of age with any stage of breast cancer. We repeated all analyses in FinnGen, which used the same sample selection as described in Mei and colleagues (15). However, even then, we could not detect a significant association of PCSK9 levels with BCS.

Third, the effect could be modified by LDL-C. Other studies have already shown that PCSK9 affects metastasis in mice and pancreatic cancer (16, 17). However, their reported effect had been dependent on cholesterol and lipid metabolism. Contrary to that, Mei and colleagues (15) reported a cholesterol-independent effect of PCSK9 on BCS. In the univariable MR approach, we were only able to estimate the total PCSK9 effect. Using MVMR, we attempted to correct for a potential LDL-C effect on BCS and to obtain the direct effect of PCSK9. Here, the direction of the effect of PCSK9 changed but remained insignificant. However, the effects at *PCSK9* are highly correlated, and the power to detect effects in the MVMR was limited: the conditional F-statistics were 9.2 and 9.3, whereas in the MR approach, we had F-statistics of 120.5 and 393.5 for PCSK9 and LDL-C, respectively.

Finally, the association in Mei and colleagues (15) may have been a false-positive finding. Although the SNP effect was validated in mouse knock-in studies, it was not replicated in the larger datasets of the BCAC (20) or FinnGen (21). It is possible that PCSK9 inhibitors might have a beneficial effect in mouse models, cell lines, or high-risk patients with breast cancer, but the generalization to all patient groups might not be valid.

To ensure our instruments and exposures were well defined, we ran positive and negative controls. For breast cancer risk, we did not expect to find any effect of PCSK9 or LDL-C using instruments at *PCSK9* or genome-wide (8, 10), and indeed, we found none. The positive controls were CAD risk in a sex-combined and a female-only dataset, and the PCSK9 effects were, according to expectation, positive on CAD. Of note, it did not matter whether we looked at rs562556 or multiple instruments or if we looked at protein or gene expression levels; the effect on sex-combined CAD risk was always positive and significant. In contrast, the BCS association was only significant when using the outcome data from four small cohorts, but not when using larger cohort and consortium data.

One limitation of this study was that we could not exactly test Mei and colleagues’ (15) suggestion that PCSK9 inhibitors would be beneficial for homozygous major allele carriers, which would focus more on PCSK9 functionality rather than just PCSK9 levels. However, the SNPs used in the multiple variant approach were rather independent of rs562556, with LD *r*^2^ < 0.05 for all but two variants: rs553741 (*r*^2^ = 0.374, eQTL in liver) and rs693668 (*r*^2^ = 0.235, pQTL). Given this independence, it is rather unlikely that the effect directions will change when restricting the GWAS to the subset of samples carrying the major allele. The SNP rs693668 has been reported for sex-biased effects, with stronger effects on PCSK9 gene expression in women (35) but stronger effects on PCSK9 protein expression in men (22). The mechanism is not yet clear, although it has been hypothesized that estradiol might regulate PCSK9 expression (36). Hence, it would be of future interest to test the MR with outcome data in patients with breast cancer stratified by estrogen receptor status (ER+ vs. ER−). Another limitation is that MR tests for lifelong effects and might be of limited use for understanding drug effects. Also, we cannot exclude potential drug interactions between chemotherapy and statin treatment, which might explain the beneficial effects observed in animal models and cell lines.

In conclusion, a significant positive effect of PCSK9 on BCS could only be reproduced when using the exact same outcome dataset from Mei and colleagues (15) but not when using independent data from a consortium (20) or FinnGen (21). Although PCSK9 might be an interesting potential therapeutic target for high-risk patients with breast cancer, the generalization for other patient groups is not supported by our data and needs to be further explored.

## Supplementary Material

Figure S1 Figure S1 shows the study design flowchart.

Figure S2 Figure S2 shows the Forest Plot of the four studies reported in Mei and colleagues.

Figure S3 Figure S3 shows the Forest plot of the single variant analysis using PCSK9 gene expression as exposure.

Figure S4 Figure S4 shows the Forest Plot of the single variant analysis using LDL-C levels as exposure.

Figure S5 Figure S5 shows Scatter plot of MR-IVW estimates using PCSK9 levels in females as exposure.

Figure S6 Figure S6 shows the Forest plot of PCSK9 gene expression on BC survival using multiple variants.

Figure S7 Figure S7 shows the Forest plot of LDL-C levels on BC survival using multiple variants.

Figure S8 Figure S8 shows the Forest plots of the MVMR analyses of PCSK9 conditional on LDL-C on BC survival.

Figure S9 Figure S9 shows the Forest plot of PCSK9 or LDL-C levels on BC risk.

Figure S10 Figure S10 shows the Forest plot of PCSK9 or LDL-C levels on CAD risk.

Figure S11 Figure S11 shows the Forest plots of the MVMR analyses of LDL-C conditional on PCSK9 on CAD risk.

Table S1 Table S1 shows the genetic exposure associations used in the MR analyses.

Table S2 Table S2 shows the genetic exposure associations used in the MVMR analysis.

Table S3 Table S3 shows all genetic outcome association used in this work.

Table S4 Table S4 shows the MR results of the single variant analysis.

Table S5 Table S5 shows the MR results of the multiple SNPs analysis.

Table S6 Table S6 shows the MVMR results.

Supplemental Data Supplemental Notes includes further information on the FinnGen genotyping, imputation, ethic statement and FinnGen group collaborators.

## Data Availability

The code is available at https://github.com/pottj/PCSK9_BreastCancerSurvival. All extracted and used summary statistics are available in Supplementary Tables S1–S3. GWAS summary statistics on PCSK9 plasma levels has been contributed by Pott and colleagues and has been downloaded from https://zenodo.org/records/10600167. Genetic summary statistics on PCSK9 gene expression has been contributed by the GTEx consortium and the GTEx eQTL Browser and has been downloaded from https://console.cloud.google.com/storage/browser/gtex-resources. GWAS summary statistics on LDL-C levels has been contributed by the GLGC and has been downloaded from https://csg.sph.umich.edu/willer/public/glgc-lipids2021/results/sex_and_ancestry_specific_summary_stats/. GWAS summary statistics on BCS has been contributed by the BCAC and has been downloaded from https://www.ccge.medschl.cam.ac.uk/breast-cancer-association-consortium-bcac/data-data-access/summary-results/gwas-summary-results-0. GWAS summary statistics on breast cancer has been contributed by FinnGen and UKB and has been downloaded from https://console.cloud.google.com/storage/browser/finngen-public-data-r10/ukbb/. GWAS summary statistics on CAD has been contributed by Aragam and colleagues and has been downloaded from https://hugeamp.org/dinspector.html?dataset=Aragam2022_CAD_Mixed_females&phenotype=CAD and https://hugeamp.org/dinspector.html?dataset=Aragam2022_CAD_EU&phenotype=CAD. In FinnGen, the individual-level data are available under restricted access for legal and ethical reasons. Formal approval for the researchers is required to access the data; please see https://www.finngen.fi/en/access_results for more details. To get access to FinnGen summary statistics, fill out an online form at: https://elomake.helsinki.fi/lomakkeet/124935/lomake.html. Access to individual-level data and genotype data is managed by the Finnish Biobank Cooperative at the Fingenious portal [https://site.fingenious.fi/en/]. The expected response time for access requests to individual-level data is 1 to 2 months.
