# Supplementary material for: PCSK9 and Breast Cancer Survival: A Mendelian Randomization Study
Source: Cancer Epidemiol Biomarkers Prev. 2026 Mar 23;35(6):873–82. doi: 10.1158/1055-9965.EPI-25-1569 (PMC13227093; doi:10.1158/1055-9965.EPI-25-1569)

Figure S1: Study design flowchart. We used three exposures to test for an effect of PCSK9 on BC Survival: PCSK9 protein levels from Pott et al., PCSK9 gene expression levels from GTEx v10, and LDL-C levels from the Global Lipid Genetics Consortium. We tested three replication approaches: using only one single variant as reported in Mei et al. (orange box), using multiple variants associated with genome-wide significance ($p<5x10^{-8}$) and pairwise independent (linkage disequilibrium (LD) $r^2<0.1$, blue box). For PCSK9 protein levels and gene expression, we restricted the SNP selection to the PCSK9 gene region. For LDL-C, we also screened genome-wide, using a position-based priority pruning approach. In addition, we performed a multivariable MR approach testing PCSK9 and LDL-C together. In a sensitivity analysis, we used instruments at PCSK9 and HMGCR. Primary outcome data was taken from Mei et al. (original study), and Morra et al. and FinnGen (replication studies). As control outcomes we used coronary artery disease (CAD) and breast cancer risk.

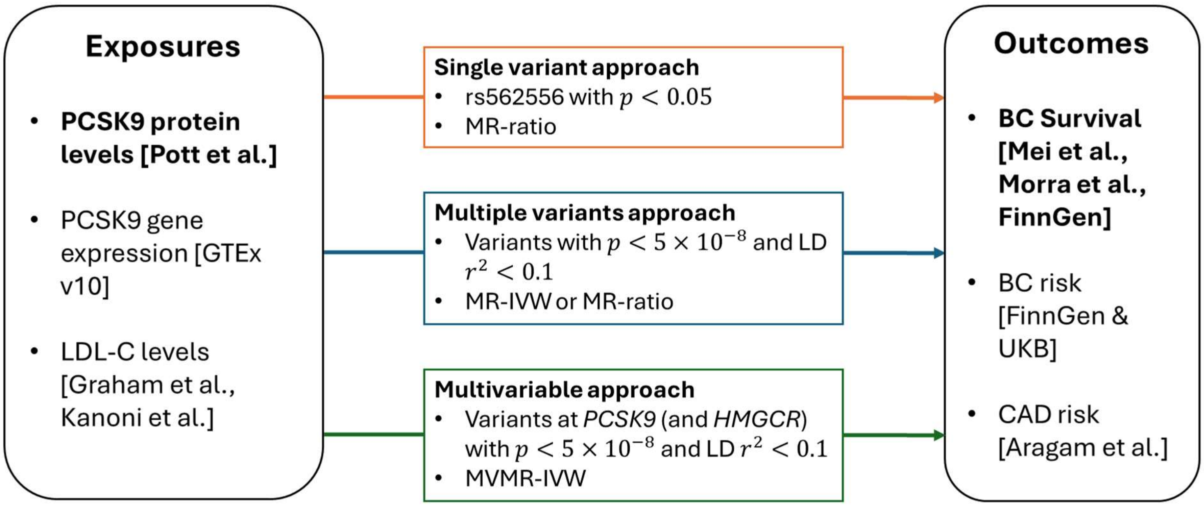

Supplement: Figure S1 — shows the study design flowchart. [file epi-25-1569_figure_s1_suppsf1.pdf]
