# Supplementary material for: PCSK9 and Breast Cancer Survival: A Mendelian Randomization Study
Source: Cancer Epidemiol Biomarkers Prev. 2026 Mar 23;35(6):873–82. doi: 10.1158/1055-9965.EPI-25-1569 (PMC13227093; doi:10.1158/1055-9965.EPI-25-1569)

Figure S2: Forest Plot of the four studies reported in Mei et al.. The summary statistic per cohort was taken from the Mei et al. Supplemental Figure S1H. Fixed-effects and random-effects meta-analysis was performed using the R-package meta. No signs of heterogeneity were detected.

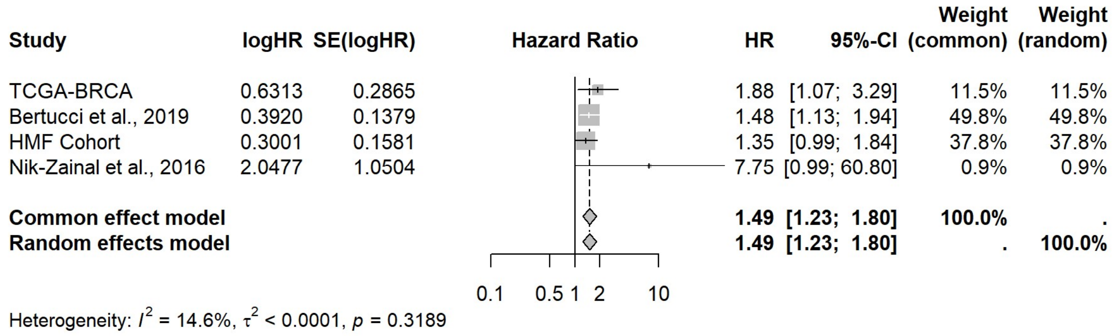

Supplement: Figure S2 — shows the Forest Plot of the four studies reported in Mei and colleagues. [file epi-25-1569_figure_s2_suppsf2.pdf]
