# Supplementary material for: PCSK9 and Breast Cancer Survival: A Mendelian Randomization Study
Source: Cancer Epidemiol Biomarkers Prev. 2026 Mar 23;35(6):873–82. doi: 10.1158/1055-9965.EPI-25-1569 (PMC13227093; doi:10.1158/1055-9965.EPI-25-1569)

Figure S3: Forest plot of the single variant analysis using PCSK9 gene expression as exposure. The log hazard ratios (logHR) for BC survival per 1 SD increment in PCSK9 gene expression (GE) levels are given for two tissues with significant eQTLs, namely spleen and esophagus muscularis. A) MR-ratio results when using the outcome data as reported by Mei et al. (their Figure S1H) and when using the fixed-effect meta-analysis estimate for the outcome. After correcting for multiple testing, only the estimates of the pooled analysis were still significant, while the effect using the Bertucci et al. study and TCGA-BRCA were not. Please note: the exact sample size per study for Europeans only was not reported. Hence we give here the sample sizes reported in Mei et al. main analysis (Figure S1C-F). B) MR-ratio results when using outcome data from the FinnGen cohort and the Breast Cancer Association Consortium (Morra et al.).

A) Original studies (Mei et al.)

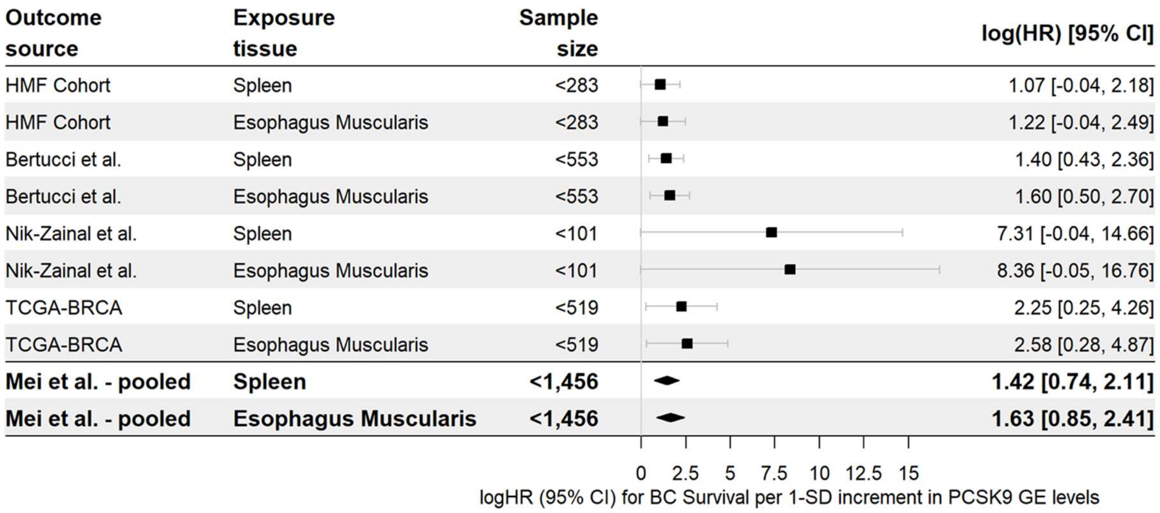

B) Replication studies

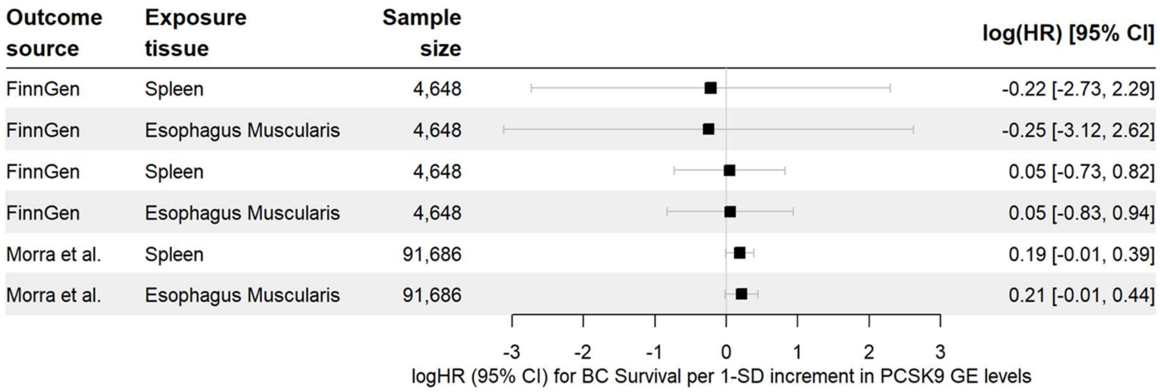

Supplement: Figure S3 — shows the Forest plot of the single variant analysis using PCSK9 gene expression as exposure. [file epi-25-1569_figure_s3_suppsf3.pdf]
