# Supplementary material for: PCSK9 and Breast Cancer Survival: A Mendelian Randomization Study
Source: Cancer Epidemiol Biomarkers Prev. 2026 Mar 23;35(6):873–82. doi: 10.1158/1055-9965.EPI-25-1569 (PMC13227093; doi:10.1158/1055-9965.EPI-25-1569)

Figure S4: Forest plot of the single variant analysis using LDL-C levels as exposure. The log hazard ratios (logHR) for BC survival per 1 SD increment in LDL-C levels are given in females only. A) MR-ratio results when using the outcome data as reported by Mei et al. (Figure S1H) and when using the fixed-effect meta-analysis estimate for the outcome. After correcting for multiple testing, only the estimates of the pooled analysis and Bertucci et al. study were still significant, while TCGA-BRCA was not. Please note: the exact sample size per study for Europeans only was not reported. Hence we give here the sample sizes reported in Mei et al. main analysis (Figure S1C-F). B) MR-ratio results when using outcome data from the FinnGen cohort and the Breast Cancer Association Consortium (Morra et al.).

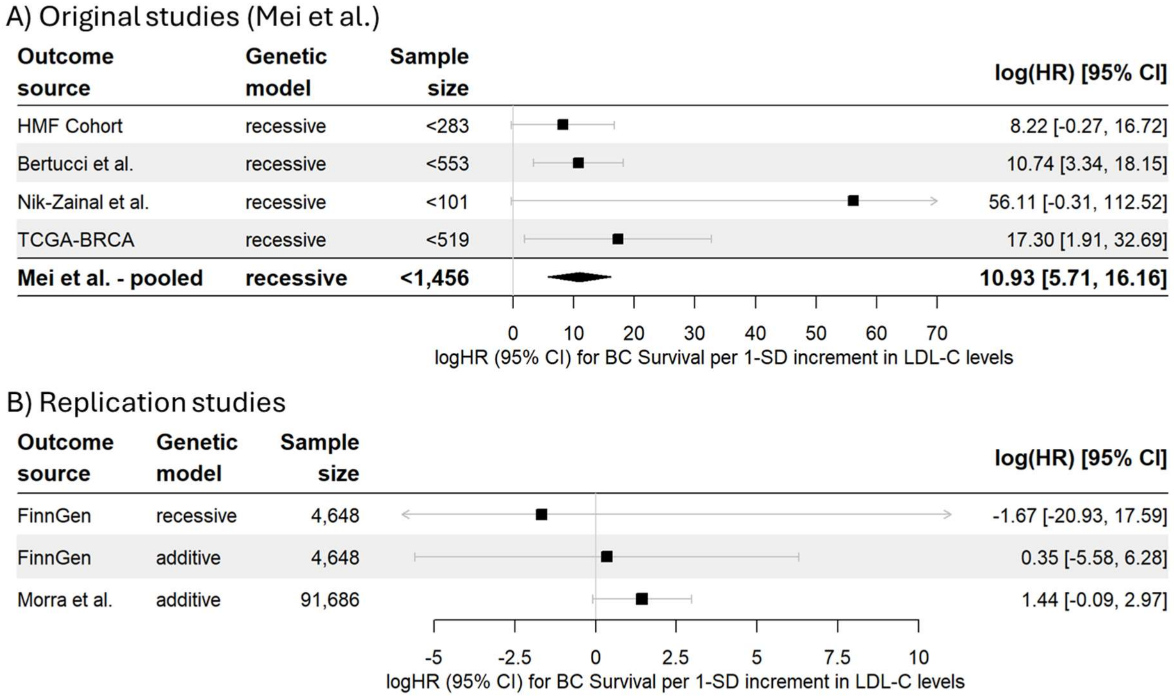

Supplement: Figure S4 — shows the Forest Plot of the single variant analysis using LDL-C levels as exposure. [file epi-25-1569_figure_s4_suppsf4.pdf]
