# Supplementary material for: PCSK9 and Breast Cancer Survival: A Mendelian Randomization Study
Source: Cancer Epidemiol Biomarkers Prev. 2026 Mar 23;35(6):873–82. doi: 10.1158/1055-9965.EPI-25-1569 (PMC13227093; doi:10.1158/1055-9965.EPI-25-1569)

Figure S5: Scatter plot of MR-IVW estimates using PCSK9 levels in females as exposure and A) BC survival (from FinnGen), B) BC survival (from Morra et al,), C) BC risk (from FinnGen & UKB(and D) Coronary Artery Disease (from Aragam et al., sex-combined) as outcome data. Error bars indicate the 95% confidence intervals of the SNP effects. The blue lines indicate the MR-IVW estimates, which were not significant for panel A – C, but significant for panel D (positive control). Estimates and F-statistics can be found in **Supplemental Table S5**.

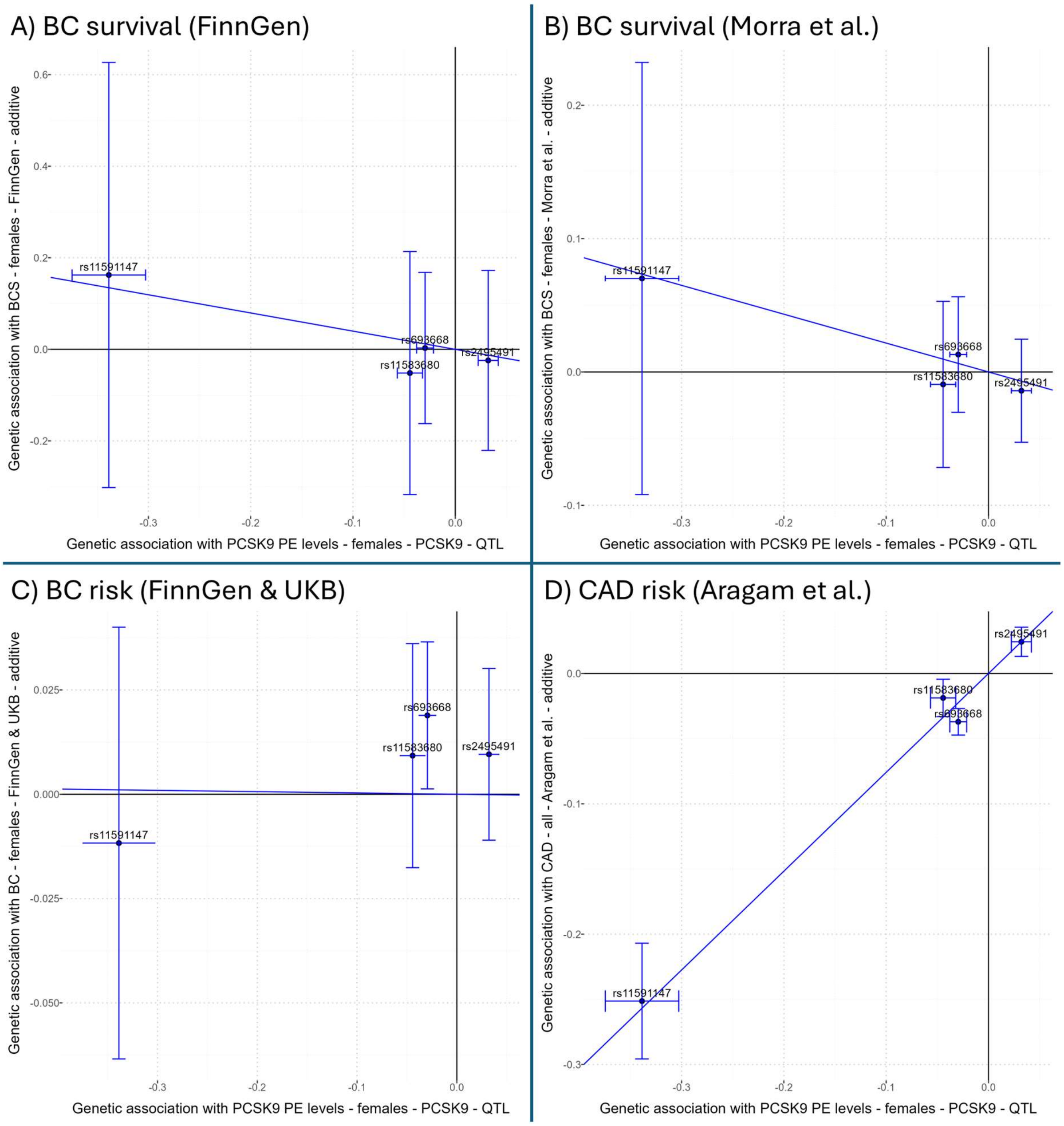

Supplement: Figure S5 — shows Scatter plot of MR-IVW estimates using PCSK9 levels in females as exposure. [file epi-25-1569_figure_s5_suppsf5.pdf]
