# Supplementary material for: PCSK9 and Breast Cancer Survival: A Mendelian Randomization Study
Source: Cancer Epidemiol Biomarkers Prev. 2026 Mar 23;35(6):873–82. doi: 10.1158/1055-9965.EPI-25-1569 (PMC13227093; doi:10.1158/1055-9965.EPI-25-1569)

Figure S6: Forest plot of PCSK9 gene expression on BC survival using multiple variants. The log hazard ratios (logHR) for BC survival per 1 SD increment in PCSK9 gene expression (GE) levels are given for selected tissues. The best eQTLs per tissue were used, either with the ratio method (only one independent eQTL) or MR-IVW (multiple independent eQTLs). We observed no significant association.

Exposure tissue	Outcome source	log(HR) [95% CI]	F-Stat
Adipose Visceral Omentum	FinnGen	-0.04 [-0.36, 0.27]	165.8
Adipose Visceral Omentum	Morra et al.	-0.03 [-0.09, 0.04]	165.8
Brain Cerebellar Hemisphere	FinnGen	0.08 [-0.32, 0.48]	82.8
Brain Cerebellar Hemisphere	Morra et al.	0.01 [-0.08, 0.11]	82.8
Brain Cerebellum	FinnGen	0.06 [-0.24, 0.36]	92.3
Brain Cerebellum	Morra et al.	0.01 [-0.06, 0.08]	92.3
Lung	FinnGen	0.23 [-0.40, 0.86]	112.9
Lung	Morra et al.	-0.06 [-0.19, 0.07]	112.9
Nerve Tibial	FinnGen	0.05 [-0.17, 0.27]	264.7
Nerve Tibial	Morra et al.	-0.03 [-0.07, 0.02]	264.7
Pancreas	FinnGen	-0.02 [-0.71, 0.67]	32.1
Pancreas	Morra et al.	0.00 [-0.15, 0.16]	32.1
Spleen	FinnGen	-0.01 [-0.59, 0.56]	41.1
Spleen	Morra et al.	-0.04 [-0.19, 0.11]	41.1
Whole Blood	FinnGen	0.14 [-0.55, 0.83]	59.9
Whole Blood	Morra et al.	-0.09 [-0.25, 0.08]	59.9

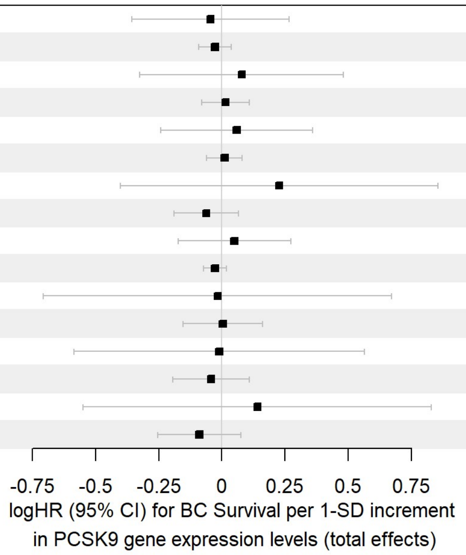

Supplement: Figure S6 — shows the Forest plot of PCSK9 gene expression on BC survival using multiple variants. [file epi-25-1569_figure_s6_suppsf6.pdf]
