# Supplementary material for: PCSK9 and Breast Cancer Survival: A Mendelian Randomization Study
Source: Cancer Epidemiol Biomarkers Prev. 2026 Mar 23;35(6):873–82. doi: 10.1158/1055-9965.EPI-25-1569 (PMC13227093; doi:10.1158/1055-9965.EPI-25-1569)

Figure S7: Forest plot of LDL-C levels on BC survival using multiple variants. The log hazard ratios (logHR) for BC survival per 1 SD increment in LDL-C levels are given for both females and sex-combined. We used the MR-IVW approach with multiple independent variants, either genome-wide (any valid SNP), at the PCSK9 gene region, or at the HMGCR gene region. We observed a significant association only for LDL-C proxied by HMGCR.

Exposure sex	Instrument selection	Outcome source	log(HR) [95% CI]	F-Stat
females	*genome-wide*	FinnGen	-0.01 [-0.32, 0.29]	400.2
sex-combined	*genome-wide*	FinnGen	-0.02 [-0.35, 0.32]	448.1
females	*genome-wide*	Morra et al.	0.01 [-0.06, 0.07]	400.2
sex-combined	*genome-wide*	Morra et al.	-0.01 [-0.08, 0.05]	448.1
females	*PCSK9*	FinnGen	-0.14 [-1.00, 0.72]	483.1
sex-combined	*PCSK9*	FinnGen	-0.20 [-1.05, 0.65]	622.3
females	*PCSK9*	Morra et al.	-0.23 [-0.48, 0.02]	483.1
sex-combined	*PCSK9*	Morra et al.	-0.20 [-0.44, 0.04]	622.3
females	*HMGCR*	FinnGen	-0.11 [-2.68, 2.47]	259.2
sex-combined	*HMGCR*	FinnGen	-0.37 [-2.40, 1.66]	289.6
females	*HMGCR*	Morra et al.	-0.51 [-0.87, -0.16]	259.2
sex-combined	*HMGCR*	Morra et al.	-0.56 [-0.93, -0.19]	289.6

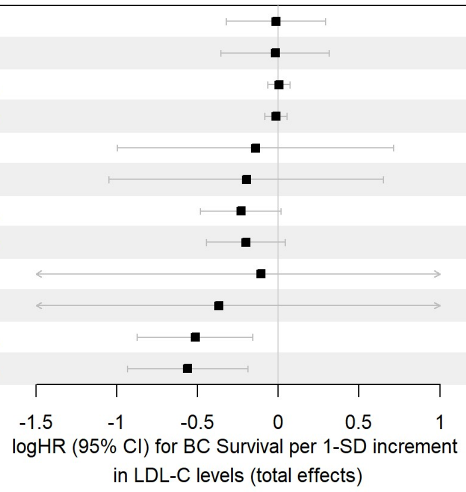

Supplement: Figure S7 — shows the Forest plot of LDL-C levels on BC survival using multiple variants. [file epi-25-1569_figure_s7_suppsf7.pdf]
