# Supplementary material for: PCSK9 and Breast Cancer Survival: A Mendelian Randomization Study
Source: Cancer Epidemiol Biomarkers Prev. 2026 Mar 23;35(6):873–82. doi: 10.1158/1055-9965.EPI-25-1569 (PMC13227093; doi:10.1158/1055-9965.EPI-25-1569)

Figure S8: Forest plots of the MVMR analyses of PCSK9 conditional on LDL-C on BC survival. The log hazard ratios (logHR) for breast cancer survival per 1 SD increment in PCSK9 or LDL-C levels are given per sex setting. We used either 20 or 35 independent variants at PCSK9 and HMGCR for females or sex-combined, respectively. Estimates and F-statistics can also be found in **Supplemental Table S6**.

Exposure	Exposure sex	Outcome source	log(HR) [95% CI]	cond. F-Stat
PCSK9	females	FinnGen	-0.60 [-3.33, 2.14]	11.1
LDL-C	females	FinnGen	0.19 [-1.53, 1.91]	11.2
PCSK9	all	FinnGen	-0.31 [-2.56, 1.94]	15.2
LDL-C	all	FinnGen	-0.05 [-1.58, 1.48]	15.2
PCSK9	females	Morra et al.	0.43 [-0.14, 1.00]	11.1
LDL-C	females	Morra et al.	-0.51 [-0.83, -0.19]	11.2
PCSK9	all	Morra et al.	0.52 [-0.03, 1.06]	15.2
LDL-C	all	Morra et al.	-0.56 [-0.89, -0.23]	15.2

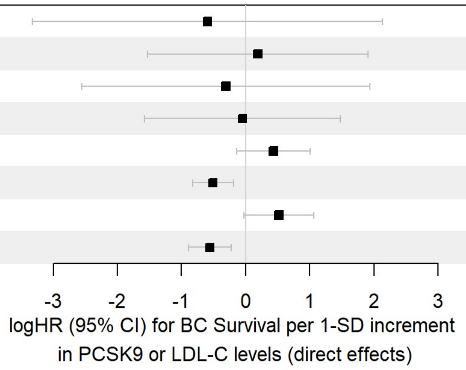

Supplement: Figure S8 — shows the Forest plots of the MVMR analyses of PCSK9 conditional on LDL-C on BC survival. [file epi-25-1569_figure_s8_suppsf8.pdf]
