# Supplementary material for: PCSK9 and Breast Cancer Survival: A Mendelian Randomization Study
Source: Cancer Epidemiol Biomarkers Prev. 2026 Mar 23;35(6):873–82. doi: 10.1158/1055-9965.EPI-25-1569 (PMC13227093; doi:10.1158/1055-9965.EPI-25-1569)

Figure S9: Forest plot of PCSK9 or LDL-C levels on BC risk. The log odds ratios (logOR) for breast cancer (BC) risk per 1 SD increment in PCSK9 or LDL-C levels are given for respective sex setting. BC was our negative control outcome, and indeed the estimates are not significant in all tests. A) Results using the single variant approach testing only the variant rs562556. B) Results using multiple variants at the PCSK9 gene region.

A) Single variant approach (rs562556) on BC risk

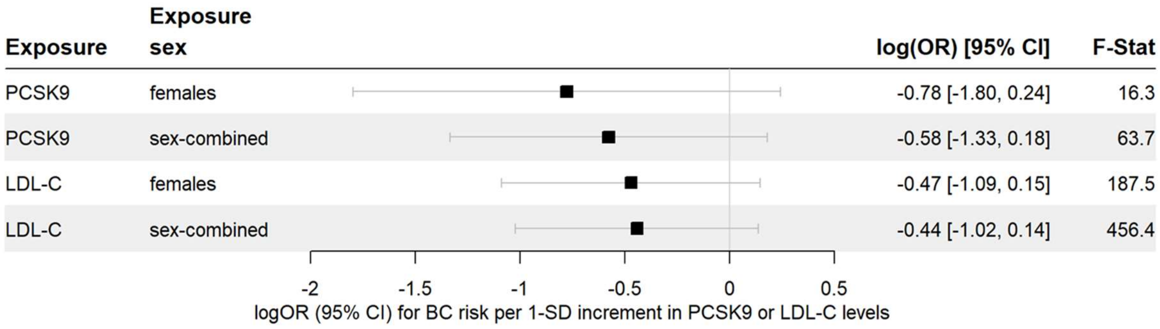

B) Multiple variant approach on BC risk

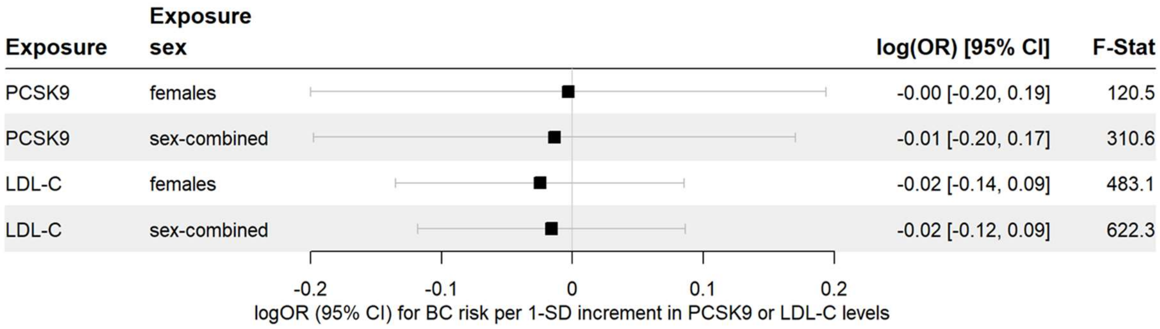

Supplement: Figure S9 — shows the Forest plot of PCSK9 or LDL-C levels on BC risk. [file epi-25-1569_figure_s9_suppsf9.pdf]
