# Supplementary material for: PCSK9 and Breast Cancer Survival: A Mendelian Randomization Study
Source: Cancer Epidemiol Biomarkers Prev. 2026 Mar 23;35(6):873–82. doi: 10.1158/1055-9965.EPI-25-1569 (PMC13227093; doi:10.1158/1055-9965.EPI-25-1569)

Figure S10 Forest plot of PCSK9 or LDL-C levels on CAD risk. The log odds ratios (logOR) for coronary artery disease (CAD) risk per 1 SD increment in PCSK9 or LDL-C levels are given for respective sex setting. CAD was our positive control outcome, and indeed all estimates are significant after multiple testing correction. A) Results using the single variant approach testing only the variant rs562556. B) Results using multiple variants at the PCSK9 gene region.

A) Single variant approach (rs562556) on CAD risk

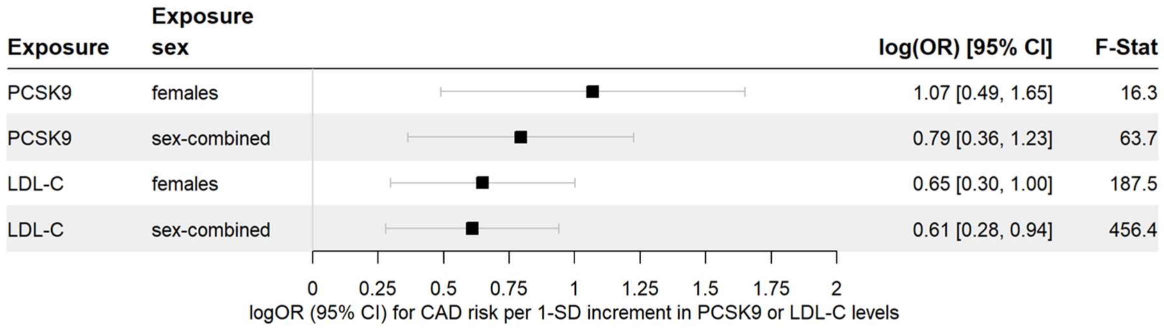

B) Multiple variant approach on CAD risk

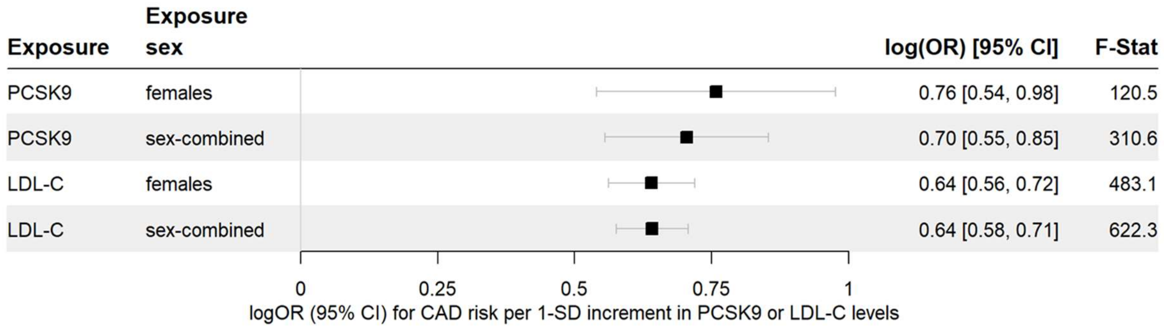

Supplement: Figure S10 — shows the Forest plot of PCSK9 or LDL-C levels on CAD risk. [file epi-25-1569_figure_s10_suppsf10.pdf]
