# Supplementary material for: PCSK9 and Breast Cancer Survival: A Mendelian Randomization Study
Source: Cancer Epidemiol Biomarkers Prev. 2026 Mar 23;35(6):873–82. doi: 10.1158/1055-9965.EPI-25-1569 (PMC13227093; doi:10.1158/1055-9965.EPI-25-1569)

Figure S11: Forest plots of the MVMR analyses of LDL-C conditional on PCSK9 on CAD risk. The log odds ratios (logOR) for coronary artery disease per 1 SD increment in PCSK9 or LDL-C levels are given per exposure sex setting. A) MVMR results using four independent variants at PCSK9. B) MVMR results using 20 or 35 independent variants at PCSK9 and HMGCR for females and sex-combined, respectively.

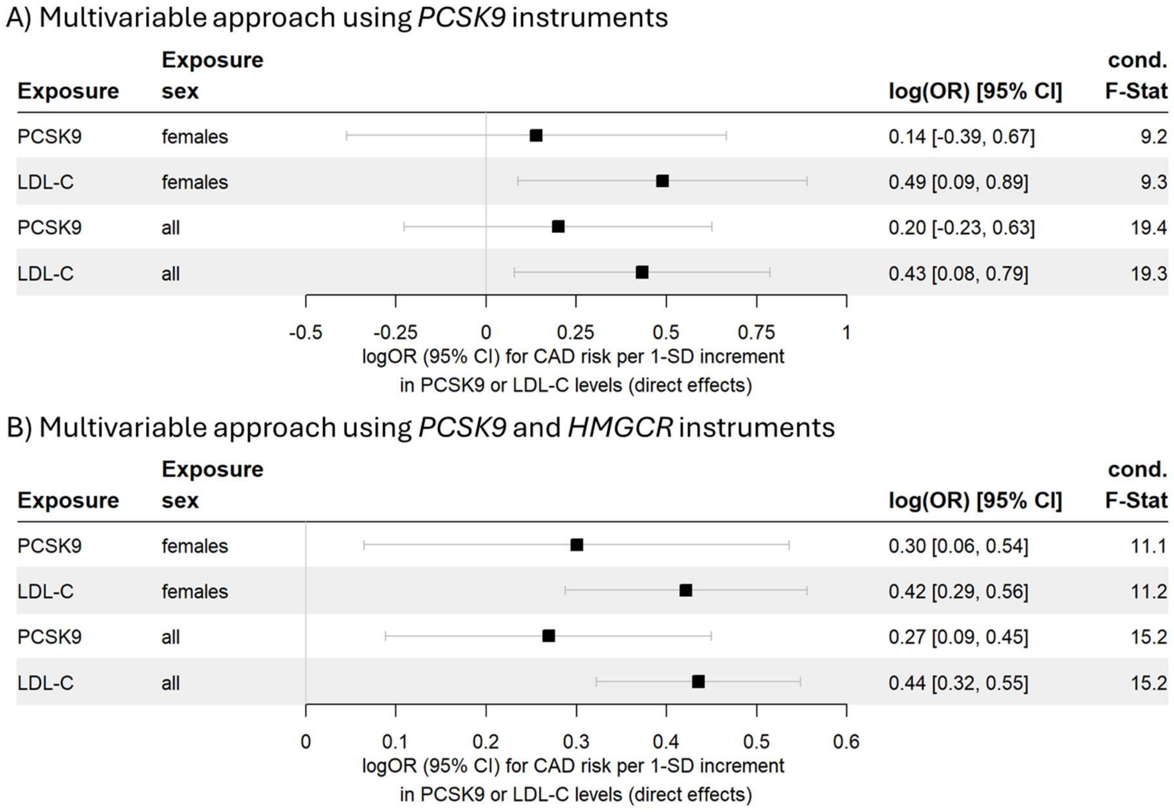

Supplement: Figure S11 — shows the Forest plots of the MVMR analyses of LDL-C conditional on PCSK9 on CAD risk. [file epi-25-1569_figure_s11_suppsf11.pdf]
